# Role of sulphated polysaccharides from *Sargassum Wightii *in Cyclosporine A-induced oxidative liver injury in rats

**DOI:** 10.1186/1471-2210-8-4

**Published:** 2008-02-20

**Authors:** Anthony Josephine, Kalaiselvam Nithya, Ganapathy Amudha, Coothan Kandaswamy Veena, Sreenivasan P Preetha, Palaninathan Varalakshmi

**Affiliations:** 1 Department of Medical Biochemistry, Dr. ALM. Post Graduate Institute of Basic Medical Sciences, University of Madras, Taramani Campus, Chennai – 600 113, India

## Abstract

**Background:**

Seaweeds or marine algae have long been made up a key part of the Asian diet, and as an antioxidant, sulphated polysaccharides have piqued the interest of many researchers as one of the ocean's greatest treasures. The present investigation suggests the therapeutic potential of sulphated polysaccharides from marine brown algae "*Sargassum wightii*" in Cyclosporine A (CsA)- induced liver injury. CsA is a potent immunosuppressive agent used in the field of organ transplantations and various autoimmune disorders. However, hepatotoxicity due to CsA remains to be one of the major clinical challenges.

**Methods:**

The effect of sulphated polysaccharides on CsA-induced hepatotoxicity was studied in adult male albino rats of Wistar strain, and the animals were randomized into four groups with six rats in each. Group I served as vehicle control. Group II rats were given CsA at a dosage of 25 mg/kg body weight, orally for 21 days. Group III rats were given sulphated polysaccharides at a dosage of 5 mg/kg body weight, subcutaneously for 21 days. Group IV rats were given sulphated polysaccharides simultaneously along with CsA, as mentioned in Group II for 21 days.

**Results:**

CsA provoked hepatotoxicity was evident from the decreased activities of hepatic marker enzymes. A significant rise in the level of oxidants, along with a striking decline in both the enzymic and non-enzymic antioxidants, marks the severity of oxidative stress in CsA-induced rats. This in turn led to enhanced levels of lipid peroxidation, 8-hydroxy-2-deoxy guanosine and protein carbonyls, along with a decrease in ATPase activities and alterations in lipid profile. Histopathological changes also strongly support the above aberrations. However, concomitant treatment with sulphated polysaccharides restored the above deformities to near control and prevented the morphological alterations significantly.

**Conclusion:**

Thus, the present study highlights that sulphated polysaccharides can act therapeutically against CsA-induced hepatotoxicity.

**Key Words:**

Cyclosporine A; hepatic markers; antioxidants; hyperlipidemia; macromolecules; sulphated polysaccharides.

## Background

The brown algae harvested from the earth's ocean are truly one of the most valuable gifts of the great deep. *Sargassum wightii *is one of the marine brown algal species widely found in India, with tremendous biological applications and are known to be rich in sulphated polysaccharides content. Sulphated polysaccharides were found to possess wide pharmacological actions, especially potent free radical scavenging [1] and antioxidant [2] effects. Furthermore, Raghavendran et al. [3] have documented that the hot water extract of *Sargassum polycystum *was proved to be an effective hepatoprotective agent against acetaminophen-induced liver damage. Further, Wong et al. [4] have tested the effects of three seaweeds, and found that *Sargassum henslowianum *exhibited significant protection against carbon tetrachloride-induced liver injury in rats, by reducing the acute increase of glutamate oxaloacetate transaminase (GOT) and glutamate pyruvate transaminase (GPT) levels.

The introduction of Cyclosporine A (CsA) to the transplant community had great significance, primarily because of the improved graft survival rates with corresponding decrease in rate of rejection episodes, due to its immunosuppressive action. CsA also finds application in the management of various autoimmune diseases. However, its clinical and experimental use is hampered by several side effects, among which, hepatotoxicity is also one of the most disquieting side effects [5]. Cholestasis, hyperbilirubinemia, hypoproteinemia, increased alkaline phosphatase and transaminases activities, bile salts in the blood, inhibition of protein synthesis and disturbed lipid secretion in both human and experimental animals was found to characterize CsA-induced hepatotoxicity [5,6]. Additionally, alterations in bile formation, the capacity of the liver to excrete organic anions and xenobiotics and changes in the hepatic content of glutathione (GSH) was also prominent in CsA provoked liver damage [7]. Although, various mechanisms for CsA-induced hepatotoxicity have been proposed, the precise molecular mechanism still remains a matter of disagreement. Nevertheless, numerous current findings suggest that reactive oxygen species (ROS) production, oxidative stress, depletion of hepatic antioxidant system and increase in malondialdehyde (MDA) are the possible mechanisms of CsA-induced hepatotoxicity [8].

Further, there are evidences suggesting that antioxidants could play a beneficial role in CsA- induced hepatotoxicity. It has been demonstrated that the hepatoprotective activity of the marine algal extracts may also possibly due to their antioxidant properties; acting as scavengers of free radicals such as superoxide and alkoxy radicals [9]. Hence, these findings created a background and interested us to test whether the administration of sulphated polysaccharides from *Sargassum wightii *could have any role on CsA-induced hepatotoxicity.

## Methods

### Drugs and Chemicals

Cyclosporine A (CsA) was procured from Sandoz Ltd, Basel, Switzerland. Bovine serum albumin and 1, 1', 3, 3'-tetraethoxypropane were obtained from Sigma Chemicals, St. Louis, MO, USA. All other chemicals and solvents used were of analytical grade.

### Seaweed collections and processing

The marine brown algal species *Sargassum wightii *was collected from Mandapam, Gulf of Mannar region, Rameswaram, India. The seaweed sample was washed in seawater and fresh water thoroughly to remove the contamination. The sample was then air dried in shade and coarsely powdered and used for the further isolation procedure. The extraction procedure was carried out according to the method of Vieira et al. [10], as previously described [11]. Our earlier report [12] suggests that the sulphated polysaccharides extracted from *Sargassum wightii *contain the following components: Total sugar (69.26%), protein (3.01%), uronic acid (15.62%), sulphate (12.09%) and sulphated glycosaminoglycans (74.21%).

### Animal model

Adult male albino rats of Wistar strain (180 ± 20 g) were purchased from Tamil Nadu Veterinary and Animal Sciences University, Chennai, India. The animals were maintained under standard conditions of humidity, temperature (25 ± 2°C) and 12 h light/12 h dark phases. They were fed standard rat pelleted diet (M/s Pranav Agro Industries Ltd., India) under the trade name Amrut rat/mice feed and had free access to water. Experimental animals were handled according to the University and Institutional Legislation, regulated by the committee for the purpose of Control and Supervision of Experiments on Animals (CPCSEA), Ministry of Social Justice and Empowerment, Government of India (IAES No. 02/052a/06).

The animals were divided into four groups of six rats each as follows. Group I served as olive oil treated vehicle control. Group II were administered CsA (25 mg/kg body weight, orally) for 21 days. Group III were given sulphated polysaccharides (5 mg/kg body weight, subcutaneously) for 21 days as drug control. Group IV rats were treated with sulphated polysaccharides (5 mg/kg body weight) concomitantly along with CsA induction (25 mg/kg body weight) for 21 days.

At the end of the experimental period, all the animals were sacrificed by cervical decapitation. Liver tissues were immediately excised and rinsed in ice-cold physiological saline. The tissues were homogenized in 0.01 M Tris-HCl buffer, pH 7.4. Aliquots of the tissue homogenate were suitably processed for the assessment of following biochemical parameters; also a section was set aside for histological processing.

### Biochemical assays

Aspartate aminotransferase (AST) and alanine aminotransferase (ALT) were assayed in the liver and their activities were expressed in terms of μmoles of pyruvate liberated/min/mg protein at 37°C [13]. Alkaline phosphatase (ALP) activity was estimated by using the standard protocol [14] and the enzyme activity was expressed in terms of μmoles of phenol liberated/min/mg protein. Activity of lactate dehydrogenase (LDH) was determined by using the standardized procedure [15]. Protein content was monitored by the method of Lowry et al. [16].

### Evaluation of oxidative stress and hepatic antioxidant status

Oxidants generation in hepatic tissue was assessed using the fluorescent probe, 2', 7' – dichlorodihydrofluorescein-diacetate (DCFH-DA) [17]. The enzyme superoxide dismutase (SOD) was assayed according to the method of Marklund and Marklund [18]. The unit of enzyme activity is defined as the amount of enzyme required to give 50% inhibition of pyrogallol auto-oxidation. The activity of catalase (CAT) in the tissue homogenate was assessed by the method of Sinha [19]. Glutathione peroxidase (GP_X_) was assayed according to the method of Rotruck et al. [20] and its activity was assessed in terms of utilization of glutathione. Total reduced glutathione (GSH) was estimated by the method of Moron et al. [21]. This method was based on the reaction of reduced glutathione with 5, 5'dithio-bis (2-nitrobenzoic acid) (DTNB) to give a compound that absorbs at 412 nm. Vitamin C was assayed by the procedure of Omaye et al. [22] and Vitamin E was quantified in the tissues using the method of Desai et al. [23].

### Assessment of cellular macromolecular damage

Lipid peroxidation (LPO) in the liver tissue was determined by the method of Hogberg et al. [24]. Malondialdehyde (MDA), formed as an end product of the peroxidation of lipids, served as an index of the intensity of oxidative stress. MDA reacts with thiobarbituric acid to generate a coloured product, which absorbs at 532 nm.

8-hydroxy-2-deoxyguanosine (8-OHdG) was quantified by High performance liquid chromatography (HPLC) using the standardized procedure [25]. Initially, the nuclear DNA was isolated from the liver by the method of Gross-Bellard et al. [26]. DNA thus isolated was dissolved in 20 mM acetate buffer (pH 5.0) and digested to deoxynucleosides with nuclease P_1 _and with alkaline phosphatase at 37°C for 1 h in 0.1 M Tris-HCl buffer (pH 7.5). This was then followed by the injection of the deoxynucleosides onto a C_18 _column equipped with both UV and electrochemical detectors. The level of 8-OHdG was determined based on the peak height of authentic 8-OHdG with electrochemical detector and the UV absorbance at 254 nm.

Protein carbonyl content in the tissue homogenate was determined by the method based on the reaction of carbonyl groups with 2, 4-dinitrophenyl hydrazine (DNPH) to form 2, 4-dinitrophenylhydrazone [27].

### Assessment of tissue membrane integrity

Na^+^, K^+^-Adenosine triphosphatase (Na^+^, K^+^-ATPase) was estimated by the method of Bonting [28]. The method of Hjerten and Pan [29] and Ohnishi et al. [30] was followed for the assay of Ca^2+^-Adenosine triphosphatase (Ca^2+^-ATPase) and Mg^2+^-Adenosine triphosphatase (Mg^2+^-ATPase). The amount of phosphorus liberated was determined by the method of Fiske and Subbarow [31]. Enzymic activities were expressed as μmoles of Pi liberated/min/mg protein.

### Analysis of lipid profile

Lipids were extracted from the liver tissues according to the method of Folch et al. [32] using chloroform-methanol mixture (2:1 v/v). Cholesterol estimation was done by the method of Parekh and Jung [33] using ferric acetate – uranyl acetate as the chromogenic reagent. Phospholipids were determined by the method of Rouser et al. [34]. Triglycerides and free fatty acid were quantified colorimetrically [35,36].

### Histopathological analysis

A portion of the liver tissue, immediately after sacrifice was kept in 10% formalin to fix the tissue. The tissues were washed in running tap water, dehydrated in the descending grades of isopropanol and finally cleared in xylene. The tissues were then embedded in molten paraffin wax. Sections were cut at 10 μm thickness, stained with haematoxylin and eosin. The sections were then viewed under light microscope for histopthological changes.

### Statistical analysis

The values are expressed as mean ± standard deviation (S.D) for six animals in each group. Differences between groups were assessed by one-way analysis of variance (ANOVA) using SPSS software package for Windows. Post hoc testing was performed for inter-group comparisons using the least significance difference (LSD) test; significance at *p*-value (<0.001, <0.01, < 0.05) have been given respective symbols in the tables and figures.

## Results

### Hepatic marker enzymes

Induction of CsA provoked severe biochemical amendments as well as oxidative imbalance in hepatic tissues. Table 1 displays the abnormally declined activities of hepatic enzymes that serve as a hallmark of liver damage provoked by CsA. Activities of these marker enzymes were almost restored to near control values (*P *< 0.001 and *P *< 0.01) by sulphated polysaccharides co-administration.

**Table 1:** Effect of CsA and sulphated polysaccharides on hepatic marker enzymes

Enzyme parameters	Group I (Control)	Group II (CsA)	Group III (Sulphated polysaccharides)	Group IV (CsA + sulphated polysaccharides)
AST	0.22 ± 0.02	0.14 ± 0.01 a***	0.23 ± 0.03	0.19 ± 0.02 a*b***
ALT	0.18 ± 0.02	0.09 ± 0.01 a***	0.17 ± 0.02	0.15 ± 0.02 a*b***
LDH	11.57 ± 1.40	6.83 ± 0.75 a***	11.52 ± 1.13	10.22 ± 1.23 b***
ALP	1.58 ± 0.19	1.25 ± 0.15 a**	1.60 ± 0.19	1.54 ± 0.15 b**

Values are expressed as mean ± S.D. for six animals in each group.

Units: AST, ALT – μmoles × 10^-2 ^of pyruvate liberated/min/mg protein; LDH – μmoles × 10^-1 ^of pyruvate liberated/min/mg protein; ALP – μmoles × 10^-2 ^of phenol liberated/min/mg protein.

Comparisons are made between: a – Group I and Groups II, III, IV; b – Group II and Group IV. The symbols (***), (**) and (*) represent statistical significance at *P *< 0.001, *P *< 0.01 and *P *< 0.05, respectively.

### Oxidative stress and its impact on macromolecules

**Figure 1:**
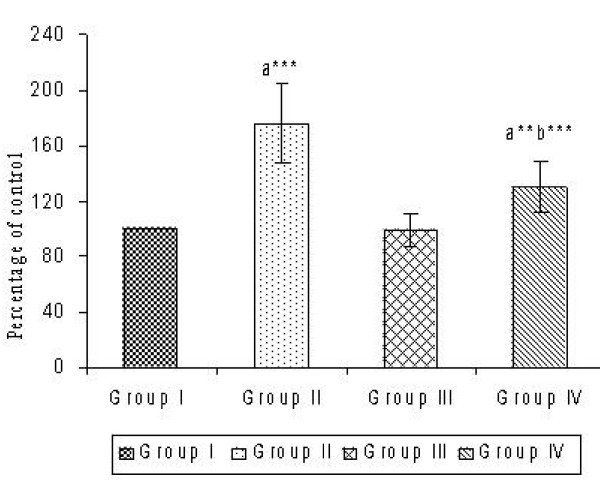
Effect of CsA and sulphated polysaccharides on hepatic oxidants production. Values are expressed as mean ± S.D. for six animals in each group. Group I – Control; Group II – CsA; Group III – Sulphated polysaccharides; Group IV – CsA+sulphated polysaccharides. Comparisons are made between: a-Group I and Groups II, III, IV; b-Group II and Group IV. The symbols (***) and (**) represent statistical significance at *P *< 0.001 and *P *< 0.01, respectively.

Figure 1 portrays the extent of oxidants generation during CsA administration and the protective effect of sulphated polysaccharides in reducing the level of oxidants. Table 2 presents the status of antioxidant defense system. Statistically significant (*P *< 0.001 and *P *< 0.01) decrease in the activities of antioxidant enzymes (SOD, CAT and GPx) and the level of non-enzymic antioxidants (GSH, vitamin C and vitamin E) were observed in CsA induced Group II animals. This enzyme inhibition was markedly prevented upon concomitant administration of sulphated polysaccharides (SOD – 1.31-fold, CAT – 1.27-fold, GPx – 1.46-fold) and improved the non-enzymic antioxidants level (GSH – 1.31-fold, Vitamin C – 1.33-fold, Vitamin E – 1.41-fold) in the CsA given animals. The inhibition of oxidants production and restoration of enzymic and non-enzymic antioxidant status within control limits by sulphated polysaccharides indicates its potent antioxidant effect against oxidative stress induced by CsA.

**Table 2:** Effect of CsA and sulphated polysaccharides on the antioxidant defense system in the hepatic tissues

Antioxidants	Group I (Control)	Group II (CsA)	Group III (Sulphated polysaccharides)	Group IV (CsA + sulphated polysaccharides)
**Enzymic antioxidants**				
SOD	8.73 ± 0.96	6.52 ± 0.79 a***	8.75 ± 0.87	8.54 ± 0.94 b***
CAT	342.67 ± 41.40	260.50 ± 28.63 a***	341.92 ± 41.31	329.84 ± 32.65 b**
GPx	18.76 ± 1.86	12.38 ± 1.63 a***	19.01 ± 2.09	18.12 ± 1.79b***
**Non-enzymic antioxidants**				
GSH	16.89 ± 1.86	12.53 ± 1.24 a***	16.82 ± 2.00	16.47 ± 1.81 b***
Vitamin C	1.92 ± 0.21	1.26 ± 0.15 a***	1.96 ± 0.19	1.67 ± 0.20 a*b**
Vitamin E	0.84 ± 0.08	0.56 ± 0.07 a***	0.89 ± 0.09	0.79 ± 0.08 b***

Values are expressed as mean ± S.D. for six animals in each group.

Units of enzyme activity: SOD: Units/mg protein, one unit is equal to the amount of enzyme that inhibits the auto-oxidation reaction by 50%; CAT: μmoles of H_2_O_2 _consumed/min/mg protein; GPx: μg of reduced GSH consumed/min/mg protein; GSH, vitamin C and vitamin E: μg/mg protein.

Comparisons are made between: a-Group I and Groups II, III, IV; b-Group II and Group IV. The symbols (***), (**) and (*) represent statistical significance at *P *< 0.001, *P *< 0.01 and *P *< 0.05, respectively.

Table 3 delineates the levels of LPO, 8-OHdG and protein carbonyls in the control and experimental animals. The hepatic tissue of CsA treated rats showed a remarkable increase in LPO, 8-OHdG and protein carbonyls, when compared with the control. Concomitant treatment of rats with sulphated polysaccharides significantly (*P *< 0.001) reduced the elevated levels of LPO, 8-OHdG and protein carbonyl close to that of controls, thereby strongly confirming its potential in inhibiting the oxidation of macromolecules like lipids, DNA and proteins.

**Table 3:** Effect of CsA and sulphated polysaccharides on the levels of LPO, 8-OHdG and protein carbonyls

Parameters	Group I (Control)	Group II (CsA)	Group III (Sulphated polysaccharides)	Group IV (CsA + sulphated polysaccharides)
LPO	1.42 ± 0.14	3.89 ± 0.43 a***	1.38 ± 0.17	1.75 ± 0.17 a*b***
8-OHdG	11.59 ± 1.04	21.25 ± 3.24 a***	11.48 ± 1.26	15.76 ± 2.25 a**b***
Protein carbonyls	1.76 ± 0.16	3.38 ± 0.41 a***	1.73 ± 0.19	2.12 ± 0.26 a*b***

Values are expressed as mean ± S.D. for six animals in each group.

Units: LPO: nmoles of MDA formed/min/mg protein; 8-OHdG: fmoles of 8-OHdG/μg of nuclear DNA; Protein carbonyls: μg/mg protein.

Comparisons are made between: a-Group I and Groups II, III, IV; b-Group II and Group IV. The symbols (***), (**) and (*) represent statistical significance at *P *< 0.001, *P *< 0.01 and *P *< 0.05, respectively.

### Membrane integrity and altered lipid profile

**Figure 2:**
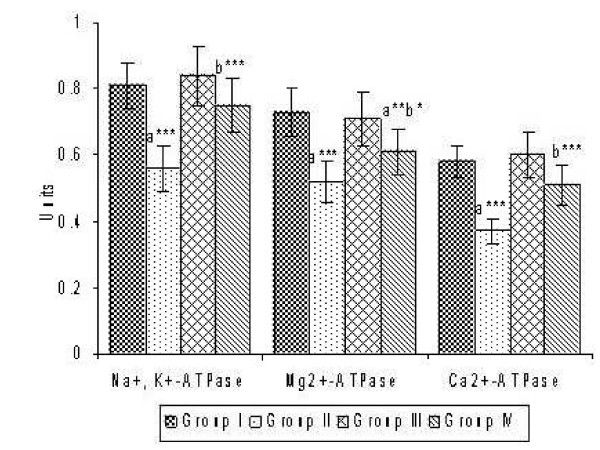
**Effect of CsA and Sulphated polysaccharides on the activities of ATPases in the liver tissues**. Values are expressed as mean ± S.D. for six animals in each group. Units: Na^+ ^K^+^-ATPase, Ca^2+^-ATPase and Mg^2+^-ATPase: μmole of inorganic phosphorous liberated/min/mg protein. Group I – Control; Group II – CsA; Group III – Sulphated polysaccharides; Group IV – CsA+sulphated polysaccharides Comparisons are made between: a-Group I and Group II, III, IV; b-Group II and Group IV. The symbols (***), (**) and (*) represent statistical significance at *P *< 0.001, *P *< 0.01 and *P *< 0.05, respectively.

Figure 2 indicates the effect of CsA and sulphated polysaccharides on ATPases activity. A significant drop in the activities of Na^+ ^K^+^-ATPase, Ca^2+^-ATPase and Mg^2+^-ATPase by 30.86%, 28.77% and 36.21% were noted in the hepatic tissue of CsA given rats. Sulphated polysaccharides co-administration restored the activities of these ATPases near to that of controls, thereby showing its beneficial effect in maintaining the membrane integrity.

Table 4 shows the level of lipid profile in the hepatic tissue of CsA induced and sulphated polysaccharides treated groups. Simultaneous treatment of CsA-induced rats with sulphated polysaccharides favourably modulated the lipid alterations, thereby proving its hypolipidemic activity.

**Table 4:** Effect of CsA and sulphated polysaccharides on lipid profile

Lipid profile (mg/g wet tissue)	Group I (Control)	Group II (CsA)	Group III (Sulphated polysaccharides)	Group IV (CsA + sulphated polysaccharides)
Total cholesterol	5.87 ± 0.71	8.01 ± 1.22 a***	5.79 ± 0.76	6.22 ± 1.01 b**
Phospholipids	20.52 ± 2.93	14.63 ± 1.93 a***	20.59 ± 3.14	19.21 ± 1.72 b**
Triglycerides	7.93 ± 0.87	10.25 ± 1.67 a**	7.90 ± 1.05	8.21 ± 1.17 b**
Free fatty acids	3.32 ± 0.44	4.26 ± 0.65 a**	3.33 ± 0.30	3.56 ± 0.39 b*

Values are expressed as mean ± S.D. for six animals in each group.

Comparisons are made between: a-Group I and Groups II, III, IV; b-Group II and Group IV. The symbols (***), (**) and (*) represent statistical significance at *P *< 0.001, *P *< 0.01 and *P *< 0.05, respectively.

### Morphological changes

**Figure 3:**
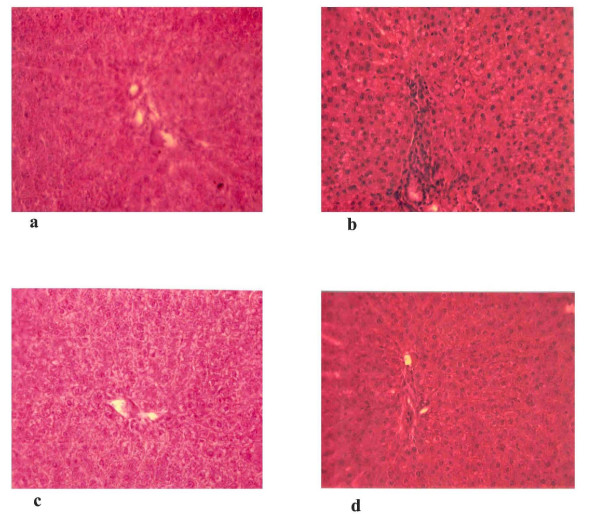
Histopathological findings in the liver tissue of CsA-induced and treated groups. Control and drug control groups show normal liver architecture (Figure 3a and 3c). Liver sections treated with CsA produces marked changes like inflammation around portal triad (Triaditis) with patchy microvesicular fatty degeneration (Figure 3b). Sulphated polysaccharides treated rats (Figure 3d) show considerable reduction in the pathological changes compared to CsA-induced animals.

Histopathological findings in liver sections (H & E, 100×) from the four experimental groups are highlighted in Figure 3. Control and drug control groups showed normal liver architecture (Figures 3a and 3c). Liver sections treated with CsA produced marked changes like inflammation around portal triad (Triaditis) with patchy microvesicular fatty degeneration (Figure 3b). Sulphated polysaccharides treated Group IV rats (Figure 3d) showed considerable reduction in the pathological changes as seen in CsA induced animals and exhibited almost normal architecture as that of controls.

## Discussion

Numerous evidences suggest that CsA-induced oxidative stress is more associated with biochemical changes that contribute to liver toxicity. It has also been reported that CsA induction results in oxidative phosphorylation uncoupling that in turn leads to the enhanced generation of free radicals [37]. Hence the role of oxidative stress has been strongly documented in the pathogenesis of CsA-induced hepatotoxicity. The present study is the first reported study of the protective role of sulphated polysaccharides on CsA-induced hepatotoxicity.

In the present study, the animals treated with CsA showed a significant liver damage, as elicited by the decreased activities of hepatic marker enzymes (AST, ALT, ALP and LDH). Decline in the activities of these hepatic enzymes also serve as an indicator of altered cell membrane permeability. It has been reported that when the liver is damaged with the introduction of infectious agents or chemicals, a significant increase in the levels of GOT and GPT were seen in the serum [38], and this increase has been attributed to damage to the structural integrity of the liver. Moreover, it has been well established that CsA administration causes a marked elevation in the activities of aminotransferases and alkaline phosphatase during hepatotoxicity [5,6], which strongly supports our present observation. LDH is the main regulator of many biochemical reactions in the body and fluids. The present study also shows a significant decrease in the activity of LDH, indicating the extent of liver damage. Administration of sulphated polysaccharides restored the activities of hepatic marker enzymes to a considerable extent. This protective action might possibly be due to its effect of preserving the cellular membrane of the hepatocytes from breakage by the reactive metabolites. Raghavendran et al. [3] have documented that the alcoholic extract of the seaweed *Sargassum polycystum *showed a potent beneficial effect against acetaminophen induced oxidative liver damage, by protecting the liver marker enzymes as well as by strengthening the antioxidant defense system.

Further, increased oxidants production in CsA-induced liver tissue was evident from DCFH oxidation. The oxidation of DCFH to the fluorescent compound DCF was considered to be a comparatively specific indicator of oxidants generation. Enormous reports suggest that ROS production, oxidative stress, depletion of hepatic antioxidant system and increase in MDA are the possible mechanisms of CsA-induced hepatotoxicity [8]. Sulphated polysaccharides co-administration minimized the oxidants production by scavenging the free radicals. Moreover, Park et al. [1] have documented that the extract from Sargassum species exerts potent free radical scavenging activity, thereby emphasizing its antioxidant potential.

It has been found that a powerful and potent endogenous antioxidant defense system functions to battle free radicals. Antioxidant enzymes such as SOD, CAT and GP_X _comprise a major supportive group of protection against free radicals. In the present investigation, a striking decrease in the activities of these antioxidant enzymes in CsA exposed rats elicits strong evidence for the involvement of oxidative damage in CsA-induced hepatotoxicity. SOD prevents the inhibition of GP_X _and CAT by scavenging superoxide radicals and GPx and CAT in turn prevent the inhibition of SOD by scavenging H_2_O_2 _[39]. Further, a marked drop in GSH levels was more significantly noted in CsA induced group. This is in line with the observation by previous reporters [7], who found that CsA induction showed a decrease in the total GSH content in the liver. Non-enzymic antioxidants further support the protective role of enzymic antioxidants, which was decreased after CsA administration in the present study. Vitamin C acts as the first line of defense in the aqueous compartment, while vitamin E is a chain breaking antioxidant, present in biological membranes. Further, the role of vitamins E and C in repairing oxidative renal toxicity after CsA administration has also been reported. Durak et al. [40] have documented that vitamins E and C combined treatment produced an increase in antioxidant capacity of hepatic tissue, which were very low in CsA treated animals.

Co-supplementation of sulphated polysaccharides was found to alleviate the oxidative injury by enhancing the antioxidant status. Sulphated polysaccharides also afforded protection by recycling both ascorbate and the membrane antioxidant vitamin E. The free radical scavenging activity of the brown algae have been tested by several authors and found that it could exhibit significant antioxidant effect by remarkably scavenging superoxide and hydroxyl radicals [41]. This possible reason can be extended to the sulphated polysaccharides from *Sargassum wightii*, in enhancing the antioxidant status during CsA induction.

The data observed in the present study thus strongly confirms that CsA-induced hepatotoxicity is associated with augmented oxidative stress. Cellular macromolecules are the important targets of ROS and their oxidative products exert diverse biological effects. This is evident from increased LPO, 8-OHdG and protein carbonyls; hallmarks of lipid, DNA and protein damage, in the CsA administered Group II animals. Oxidative destruction of polyunsaturated fatty acids (PUFA) initiates a self-perpetuating chain reaction and generation of more free radicals in a well-documented process termed, LPO, which results in loss of PUFA, and decreased membrane fluidity. There is also evidence showing that CsA increases ROS production and the formation of Thiobarbituric acid reactive substances (TBARS) in rat-cultured hepatocytes [42]. ROS also exerts adverse effects on DNA and protein by increasing the formation of 8-OHdG and protein carbonyls. The target of ROS attack could be either the sugar or the base components of the DNA molecule, and the most common base modification induced by ROS is 8-OHdG [43], which indicates DNA damage. Carbonyl content is measured as an index of protein oxidation [44], where the amino acids are converted to carbonyl derivatives. Increased protein oxidation was apparent from elevated levels of protein carbonyls in the liver tissue of CsA administered rats in the present study.

Sulphated polysaccharides co-supplementation significantly prevented the oxidative damage to macromolecules, by minimizing the levels of LPO, 8-OHdG and protein carbonyls. There is also evidence indicating that polysaccharides inhibited LPO by showing that when polysaccharides were present, the TBARS, indicative of LPO had no apparent plateau, which indicates that the formation of TBARS in the system was slowed apparently by the polysaccharides [2]. Further, the protective effect of sulphated polysaccharides against DNA damage can be positively correlated with the study of Ishikawa and Kitamura [45], which showed that heparin effectively prevents nuclear condensation and fragmentation and DNA fragmentation. These observations strongly support our study that sulphated polysaccharides could potentially inhibit cellular macromolecular damages.

It is a well-established fact that the permeability of cell membranes to various ions depends on its lipid constituents. Lipids are an essential assembly of compounds implicated in cellular function, rendering substantial contribution to the surface properties of the cell. The increased cardiovascular risk profile as a result of CsA administration is ascribed to both a quantitative increase in LDL particles and an increased oxidizability of the LDL particles [46]. Hence, knowledge of the interaction between CSA and lipid membranes is central to understanding the mechanism of its toxicity. In the present study, increase in the level of total cholesterol, triglycerides and free fatty acid in the hepatic tissue of CsA-induced rats indicates that CsA treatment might also raises the jeopardy of atherosclerosis. Princen et al. [47] have reported that there is a marked down-regulation in the activity of hepatic cholesterol 7 α-hydroxylase during CsA treatment, which might be the possible reason for the so found increased level of total cholesterol in the liver. Since, this enzyme is the rate-limiting step in cholesterol conversion to bile acid (prime pathway of cholesterol catabolism), its down-regulation by CsA therapy can lead to the accumulation of cholesterol. Phospholipids are the crucial structural components of animal cell membrane and cytoskeleton. Our data showed a decrease in tissue phospholipid content, which might be due to the increased activity of phospholipases. Further, elevations in the level of tissue triglycerides and free fatty acids indicate the liver injury due to altered lipid composition. On the other hand, co-treatment of rats with sulphated polysaccharides reduced the increased accumulation of total cholesterol and favourably modulated the lipid profiles. Enormous evidences support our observation, wherein sulphated polysaccharides from brown algae exhibit anti-hyperlipidemic effect [48], which strengthens the present observation of sulphated polysaccharides as an anti-hyperlipidemic agent.

Enhanced damages on the membrane are further evident from altered ATPases activities in the liver tissue of CsA administered groups. CsA-induced peroxidation of membrane lipids was coupled with significant inhibition of membrane ATPases. Na^+^, K^+^-ATPase is widely considered to be the enzyme system of the plasma membrane responsible for the active transport of Na^+ ^and K^+^. Ca^2+^-ATPase is responsible for the fine-tuning of intracellular calcium. Mg^2+^-ATPase also plays a role in endergonic processes other than ion transport. In the present study, reduction in the activities of ATPases in CsA administered rats may be due to the oxidation of membrane lipids and proteins. Activities of these ATPases were remarkably brought back to near control values with sulphated polysaccharides treatment, thereby maintaining the membrane integrity. Thus, sulphated polysaccharides by inhibiting the macromolecular oxidation, maintains the membrane integrity and thereby safeguards the hepatic marker enzymes.

Hepatotoxicity induced by CsA is further confirmed by abnormal histological findings. Toxicity manifestations by CsA in the liver tissue are revealed by morphological changes such as inflammation around portal triad (Triaditis) with severe patchy microvesicular fatty degeneration. Our results are in agreement with previous investigators [49], who reported morphological changes, including disorganization of hepatic parenchyma with widespread cell swelling and congestion of sinusoids during CsA administration. These changes were not observed in the rats treated with sulphated polysaccharides in Group IV animals, suggesting the protective role of sulphated polysaccharides in attenuating CsA-induced morphological changes.

## Conclusion

CsA, though it remains as a gold standard as an immunosuppressant in the transplantation field, its hepatotoxic effect remains to be one of the most tormenting side effects. The present study indicates that CsA-induced hepatotoxicity is associated with oxidative stress, cellular macromolecular damages, altered lipid profile and morphological changes. On the other hand, sulphated polysaccharides from *Sargassum wightii *were found to exhibit potent mitigating effect on oxidative liver injury induced by CsA, through its antioxidant effect, thereby signifying that sulphated polysaccharides can act as an effective hepatoprotective agent, when simultaneously given along with CsA treatment.

## Competing interests

The author(s) declare that they have no competing interests.

## Authors' contributions

KN helped in carrying out the work and analysis of the data, GA have made substantial contributions to the design of the study, CKV and SPP helped in revising it critically and PV helped in the final approval of the version to be published.
